# Visceral Metastases of Osteosarcoma in the Hepatopancreatobiliary System

**DOI:** 10.3390/jcm14248702

**Published:** 2025-12-09

**Authors:** Anna Hohensteiner, Lars Kowalscheck, Kevin Döring, Gerhard Martin Hobusch, Raphael Johannes Tanios, Oliver Strobel, Reinhard Windhager, Philipp Theodor Funovics

**Affiliations:** 1 Department of Orthopedics and Trauma-Surgery, Medical University of Vienna, 1090 Vienna, Austria; 2 Department of Pathology, Medical University of Vienna, 1090 Vienna, Austria; 3 Department of General Surgery, Medical University of Vienna, 1090 Vienna, Austria

**Keywords:** osteosarcoma, visceral metastases, pancreas, liver, follow-up

## Abstract

**Background:** Osteosarcoma (OS) is the most common primary malignant bone tumor, mainly affecting adolescents and young adults. While lung metastases are common, visceral metastases in the hepatopancreatobiliary system are extremely rare and usually associated with a poor prognosis. The limited diagnostic and therapeutic options for such metastases make the treatment of affected patients difficult. The possibility of very late metastatic onset in high-grade OS highlights the potential need for extended follow-up (FU) beyond established intervals. **Methods:** This study combines a retrospective analysis of prospectively collected data from the Vienna Bone and Soft Tissue Tumor Registry with a review of the literature of patients with OS and metastases to the hepatopancreatobiliary system. A descriptive statistical analysis is presented for the entire cohort. In addition, publications from scientific databases (PubMed, Embase) were analyzed to evaluate the frequency, diagnosis, therapy, and prognosis of visceral metastasis from both conventional OS and primary extraskeletal osteosarcoma (ESOS). **Results:** A total of six male patients with conventional OS and metastases in the liver (5) and pancreas (1), with a mean lesion size of 38 mm (range, 10–120), were included. The median age at the time of visceral metastasis was 29 years (mean, 32 years; range, 20–62 years), and the mean interval since initial diagnosis was five years and ten months (range, 9 months–10 years and 9 months). Visceral metastases are very rare in general and usually occur in advanced stages of disease. We identified 51 cases of visceral metastases from conventional OS and 34 cases of ESOS in the hepatopancreatobiliary system in the literature. The metastasis interval was three years (range, 15 months before diagnosis–17 years) at a median age of 27 years (mean, 32 years; range, 10–69 years). **Conclusions:** Visceral metastases from OS are rare but represent a significant therapeutic challenge. Early, targeted imaging in combination with improved methods for diagnosis confirmation and interdisciplinary treatment strategies may potentially improve the results. This study underlines the importance of early diagnosis and highlights the need for individualized long-term surveillance strategies exceeding ten years, especially in high-grade OS, aiming at early detection of late-onset metastasis.

## 1. Introduction

Osteosarcoma (OS) is the most common malignant bone tumor, with a peak incidence in childhood and adolescence. It has a high metastatic potential, with pulmonary lesions being the most frequent [1,2,3,4,5,6,7]. Although an increase in extrapulmonary metastasis has been observed in recent years, visceral metastases remain extremely rare [3,4,5,6,8,9,10]. Pancreatic and hepatic lesions are often asymptomatic or cause only non-specific symptoms, leading to late detection or misdiagnosis [10,11], while metastases in the duodenum can cause gastrointestinal bleeding and obstructions [3,4,12,13,14]. However, usually they occur in advanced, multimetastatic stages of disease with the patient having already developed pulmonary metastases [3,9].

Apart from visceral metastases, OS can manifest as primary extraskeletal osteosarcoma (ESOS) in the hepatopancreatobiliary system. This form of OS arises in extraosseous tissues, accounting for 1% of all soft-tissue sarcomas and 4% of osteogenic OS [15,16,17,18,19,20]. Unlike conventional OS, ESOS originates in the soft tissue without involving bone or periosteum. It exhibits a uniform sarcomatous pattern and produces an osteoid and/or cartilaginous matrix [16,19,21,22]. ESOS is both extremely rare and highly malignant, characterized by invasive growth, with a great potential for metastasis and local recurrence (LR). It predominantly affects elderly adults (average age 47.5 to 61 years), and its prognosis is poor [17,21,22,23]. Mostly, ESOS occurs in soft tissues of the limbs or the limb girdles [15,18,20]. Its involvement in visceral organs is even more uncommon, with just a few reported cases in the literature.

Regarding overall and disease-free survival, the follow-up (FU) regimen plays a crucial role in the treatment of OS, since LR and or metastatic disease are strong negative prognostic factors [24,25]. Hence, the National Comprehensive Cancer Network (NCCN) [26] recommends FU after initial treatment every three months for years 1–2, every four months for year 3, every six months until year 6, and annually thereafter. ESMO guidelines [27] and the British Sarcoma Group (BSG) [28] suggest an even stricter regimen for high-grade lesions, with a closer interval of two to four months for years 1–3, then every six months until year 5, every six to twelve months from year 5–10, and thereafter every six to twelve months. For low-grade lesions, a less frequent surveillance of every six months for years 1–2 and annually thereafter is possible. Imaging of the primary site (MRI or CT scan with contrast) and chest, as well as a complete physical exam, should be included.

This study investigates the frequency and timing of metastases in the hepatopancreatobiliary system from OS. Based on our results, we aim to infer potential implications for long-term FU, since a late metastatic onset or LR may occur more than ten years after diagnosis, and no universally accepted endpoint for surveillance currently exists.

## 2. Materials and Methods

This study combines a retrospective data analysis of patients treated for metastatic OS to the hepatopancreatobiliary system with a systematic literature review of all relevant published cases of visceral metastases of OS, as well as manifestations of ESOS in the hepatopancreatobiliary system.

All Patients of the Vienna Bone and Soft Tissue Tumor Registry with metastases to the hepatopancreatobiliary system (liver, pancreas, duodenal papilla, gall bladder) were included. Metastases were either histopathologically verified or diagnosed based on imaging, since biopsies were not performed due to advanced stages of disease. Epidemiological, clinical, radiological, and histopathological findings were extracted to investigate both demographic patient characteristics and metastatic features. The following were assessed: age (at visceral metastasis), time interval (from diagnosis to visceral involvement), lesion size, outcome (alive with disease, died of disease, died of other cause), and FU time. Overall survival of the included patients and survival after the detection of visceral metastasis were assessed using Kaplan–Meier analysis; survival curves were generated for visualization.

At our institution, FU examinations using chest and abdominal CT scans, as well as local MRI, are performed every four months during the years 1–3, subsequently every six months, and annually from year 6 onwards until year 10.

A descriptive statistical analysis (mean, range) is presented for the entire cohort. All statistical analyses were performed using Microsoft^®^ Excel macOS software (Version 16.99.2 Microsoft Corp., Redmond, WA, USA) and SPSS^®^ software (Version 29.0.2.0., SPSS Inc.: Chicago, IL, USA). Ethical approval for publication and informed consent from the patients were obtained.

For the review of the literature, case reports and series were extracted from scientific databases (PubMed and Embase) after a systematic search and were then analyzed in terms of their demographic data, treatment options, metastatic features, and outcomes. Not only histopathologically confirmed cases, but also diagnosed metastasis based on imaging or autopsies were included. Studies in languages other than English or German were translated. Case reports with limited information, as well as abstract-only publications, were excluded.

To determine the time intervals for cases with non-specific time data, estimations were inferred based on the available contextual and clinical information. Assumptions of the durations of chemotherapies (CTx) were made based on common protocols [29,30,31]: In cases of administration of adjuvant CTx, the time interval from initial diagnosis to surgical resection was considered to last three months. Full CTx (neoadjuvant and adjuvant) was estimated to last eight months, regardless of the specific protocol. FU was calculated from the first manifestation of visceral metastasis (conventional OS) or from the time of diagnosis (ESOS). Lesion size was primarily taken from reported CT imaging (largest diameter) at the time of the diagnosis. If measurements were taken from alternative imaging modalities, these values were retained, and the respective modality was specified.

## 3. Results

### 3.1. Retrospective Data Analysis

Between 1960 and December 2024, a total of 5898 patients were treated for primary bone tumors at our institution. These included 1602 malignant tumors, 1699 benign tumors, and 251 potentially malignant tumors, as well as 2346 tumor-mimicking lesions. Among all malignant bone tumors, 861 cases were identified as OS, accounting for 54% of this group. Of these, we identified and included six cases (0.7%) with metastases in the hepatopancreatobiliary system, of which only two cases (0.2%) were oligometastatic. We obtained histopathological confirmation in these two cases (patients 1 and 2). In the remaining four cases, metastasis occurred in terminal stages of disease, and no further biopsies were performed. Therefore, diagnosis in these patients is solely based on imaging findings.

**Figure 1:**
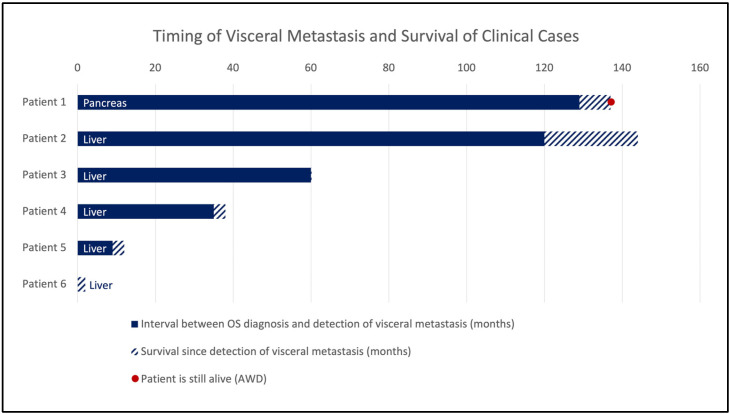
Timing of visceral metastasis after initial OS diagnosis and survival since detection of patients 1–6 (in months). Abbreviations: OS, osteosarcoma; AWD, alive with disease.

**Figure 2:**
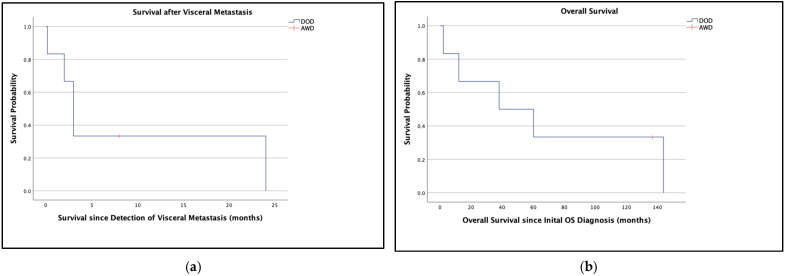
Kaplan–Meier survival analyses of patients 1–6: (**a**) Survival after detection of visceral metastasis (in months), (**b**) overall survival after OS diagnosis (in months). Abbreviations: OS, osteosarcoma; DOD, died of disease; AWD, alive with disease.

All six included patients were male and were treated for histopathologically verified conventional high-grade OS (G3). Each patient received neoadjuvant CTx, surgical resection, and adjuvant CTx according to either the EURAMOS [29] or COSS [30,31] protocol. Patients were not tested for germline syndromes, since this was not included in our standard clinical data assessment. Patient 4 (grandfather lung cancer, aunt breast cancer), patient 5 (mother brain tumor), and patient 6 (uncle pancreas carcinoma) reported a positive family history of cancer. Metastatic sites involved the pancreas in one case, and the liver in the remaining five. The median age at the time of visceral metastasis was 29 years (mean, 32 years; range, 20–62 years), and the mean interval since the initial diagnosis was five years and ten months (range, nine months–ten years and nine months). The mean overall survival was 65 months and 15 days (range, 2–144 months), and the mean survival since detection of visceral metastasis was six months and 21 days (range, five days–two years). The overall survival at five years and ten years was 50% and 33%, respectively (Figure 1 and Figure 2). One patient presented with primary lung and liver metastases. None of the patients reported symptoms directly related to pancreatic or hepatic metastases; all were asymptomatic in this regard. The mean lesion size was 38 mm. Table 1 provides a detailed overview.

In patients 3–6, visceral metastasis occurred in advanced stages, with disseminated metastases already present. No further treatment was established, and the patients died shortly thereafter. In contrast, patients 1 and 2 are to be highlighted: Each developed a solitary visceral lesion, in the pancreas and liver, respectively, as the first site of metastatic disease, more than ten years after initial diagnosis. These patients underwent a surgical resection of their metastases in terms of a curative treatment approach.

**Figure 3:**
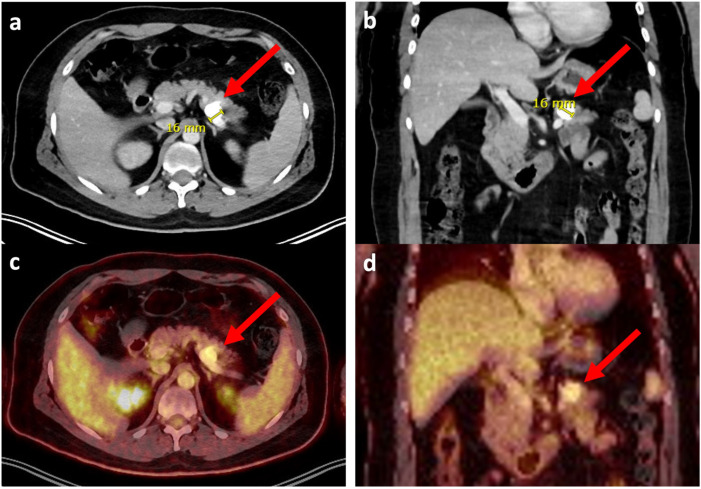
Multiplanar contrast-enhanced CT and FDG-PET/CT images demonstrating a calcified metastasis of the pancreatic tail (red arrow) in patient 1. (**a**) Axial CT showing the metastatic lesion of 11 mm extension. (**b**) Coronal CT confirming the extent and location of the lesion. (**c**) Axial fused FDG-PET/CT demonstrating increased FDG uptake corresponding to the CT-visible metastasis. (**d**) Coronal fused FDG-PET/CT further illustrates the metabolic activity of the lesion. Imaging was acquired on a Siemens Biograph 128 Vision Quadra PET-CT system using a whole-body protocol. CT acquisition was performed in the venous phase from skull to feet, with thin-slice reconstructions and evaluation in soft-tissue, lung, and bone windows. A total of 239 MBq of 18F-FDG and 80 mL of contrast agent (Iomeron 400) was administered intravenously. The dose-length product (DLP) for this examination was 1641 mGy*cm.

In contrast to patient 2, patient 1 remained free of metastasis and LR for more than ten years after the initial diagnosis and right distal femoral replacement. He was diagnosed with pancreatic tail metastasis as an incidental finding in the course of a further investigation of periprosthetic joint infection on an FDG-PET-CT scan (Figure 3). The distal femoral prosthesis was explanted and replaced with an antibiotic-enriched cement spacer. Considering a suspected pancreatic metastasis, an endosonographic biopsy was performed, but because of the calcified nature of the lesion, the sampled material was insufficient for conclusive histopathological assessment. However, a case discussion in the interdisciplinary tumor board indicated a wide resection and a minimally invasive, robotic left-sided pancreatectomy with splenectomy and gastric wedge resection was performed. The procedure was carried out within the spacer interval. Histological analysis of the resected specimen confirmed metastasis of OS with wide surgical margins. Retrospectively, a close examination of the imaging studies revealed that the lesion, first described ten months earlier at 12 mm on a CT scan and not initially considered suspicious, had increased in size by 4 mm over this period of time.

Due to the mild tumor progression and the prolonged metastasis interval of more than ten years, no systemic second-line therapy was indicated in this case. Spacer explantation and reimplantation of a new distal femoral prosthesis was performed. Nine months after diagnosis of visceral involvement, the patient remains alive and disease-free and is under close surveillance.

**Figure 4:**
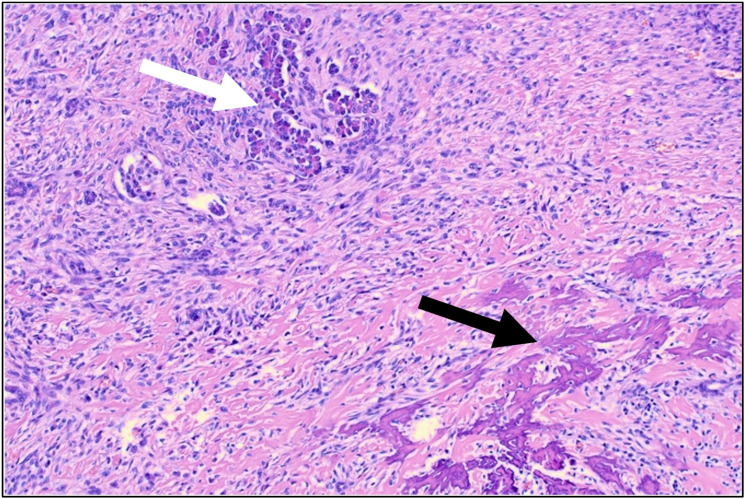
HE-stained section of calcified pancreatic metastasis from patient 1; white arrow pointing at residual pancreatic parenchyma, black arrow indicating tumor osteoid, characteristic of metastatic OS.

A further histopathological work-up was performed for patient 1 (Figure 4). While none of the suggested genetic alterations associated with an increased risk of (late-onset visceral) metastases [32] were identified, next-generation sequencing (NextSeq, Illumina, San Diego, CA, USA) using the TruSight Oncology 500 Assay (TSO500, Illumina, San Diego, CA, USA) did reveal another noteworthy finding: amplifications in MDM2 and CDK4, which may hint toward dedifferentiation of an initially low-grade central OS [33,34]. As histopathological tissue is stored for a maximum of 30 years at our institution, this work-up was not possible for the liver metastasis of patient 2. Diagnosis of visceral lesions in patients 3–6 was based on imaging; thus, no metastatic tissue is available for further investigations.

Patient 2 re-presented with recurrent fever episodes and occasional dyspnea, after being lost to FU for eight years following treatment for LR two years after the initial diagnosis. The imaging revealed a 10 mm nodule in the right lung apex on chest radiography and CT, along with a large, inhomogeneous mass in the right hepatic lobe of a 120 × 90 mm size, extending across segments V to VIII, possibly into segment IV. The liver biopsy confirmed metastatic OS. Whole-body scintigraphy showed no evidence of skeletal metastases. The patient underwent a radical right hemihepatectomy, followed by a resection of the right upper lung node. Postoperative CTx was administered, but the patient succumbed to progressive lung metastasis two years later.

### 3.2. Review of the Literature

Our systematic literature review identified 51 cases of visceral metastases from conventional OS (Table 2) and 34 cases of ESOS (Table 3) in the hepatopancreatobiliary system.

**Figure 5:**
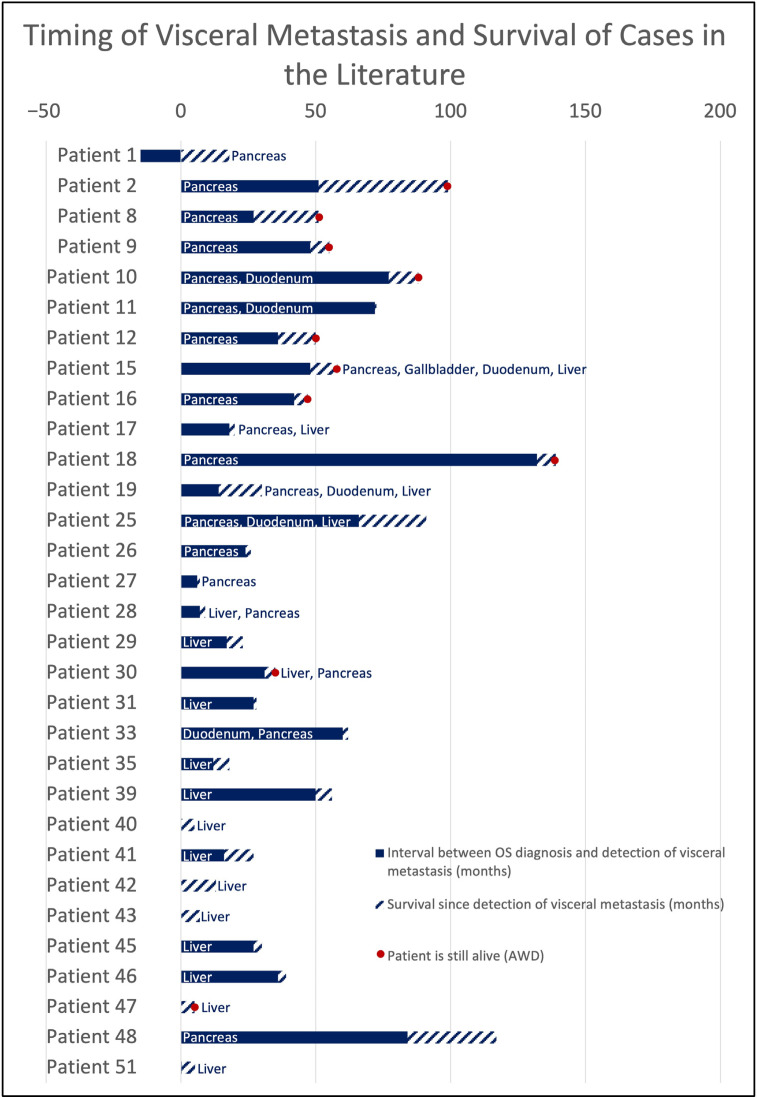
Timing of visceral metastasis and survival (in months).

Among 51 patients with visceral metastases from conventional OS, 28 were female, and 22 were male; in one case, sex was unspecified. The median age at visceral metastasis was 27 years (mean, 32 years; range, 10–69 years), with a mean interval of three years between diagnosis and occurrence of visceral metastasis (range, 15 months prior to diagnosis–17 years). The longest observed metastatic intervals were 11 [35] and 17 years [36]. Both patients already had additional metastatic sites at the time of diagnosis. The mean overall survival was 43 months (range, 3–139 months). The time intervals for patients with available data in terms of metastasis interval and survival are visualized in Figure 5.

The femur was the most affected primary tumor site (24, 47%), followed by the tibia (12, 24%). Other common sites included the humerus (4, 8%) and the fibula (3, 6%). In two cases (4%), the tumor location was not specified.

In most cases, clinical symptoms were non-specific, e.g., abdominal pain, discomfort, or distension (13, 25%). Eleven patients (22%) showed more severe gastrointestinal problems like vomiting or nausea, jaundice, diarrhea, or signs of bowel obstruction. Three patients (6%) presented with signs of gastrointestinal bleeding. Four patients (8%) were asymptomatic. Twenty cases (39%) lacked information concerning complaints.

Fifty-one metastases to the hepatopancreatobiliary system from conventional OS were distributed as follows: pancreas-only (20, 39%), liver-only (18, 35%), pancreas and liver (5, 10%), pancreas and duodenum (3, 6%), pancreas and duodenum and liver (2, 4%), duodenum-only (2, 4%), pancreas and duodenum and gall bladder (1, 2%).

As far as documented, all pancreatic metastases were solitary lesions, except for two cases. The mean size of the pancreatic lesions was 58 mm, of duodenal lesions, it was 59 mm, and of hepatic lesions, it was 44 mm. In 47% of the cases, details of the lesion’s presentation on imaging were available, with seven pancreatic metastases being calcified and four being non-calcified, as well as ten hepatic metastases being calcified and three being non-calcified.

A total of 18 patients (35%) underwent surgery as treatment for their visceral metastases, while 11 patients (22%) received (adjuvant) CTx and 3 patients (6%) had radiotherapy (RTx). Interventions like drainage of a cystic lesion, radiofrequency ablation (RFA), or antimitotic intramuscular injections were performed once each. Five patients (10%) received no further therapy due to their advanced stages. In 16 cases, treatment was not specified.

Pulmonary metastases were present in 34 cases (67%) at the time of visceral involvement (34, 67%). In eight cases (16%), visceral metastases were the initial metastatic manifestation; two of these patients subsequently developed lung metastases. However, metastases occurred only within two years after the initial diagnosis of OS in these cases.

Patients died due to advanced disease in 22 cases (43%) and due to other causes (pneumonia, sepsis, postoperative hemorrhage, and pulmonary embolism) in four cases (8%). A total of 13 patients (25%) are alive, with a maximum survival time of four years since diagnosis of visceral metastasis (mean, nine months; range, two weeks–25 months). In the remaining cases, no information was provided regarding the outcome.

We identified 34 cases of ESOS of the hepatopancreatobiliary system (Table 3). Males (20, 59%) were more often affected than females (14, 41%), and patients were significantly older than those suffering from conventional OS, with a median age of 64 years (mean, 61 years; range, 19–67 years). The liver was the most commonly affected site (23, 68%). Unlike patients with visceral metastases in conventional OS, ESOS patients were symptomatic, primarily having abdominal pain, potentially due to the larger tumor size upon diagnosis. The mean size of ESOS among all involved cases was 113 mm (range, 27–250). None of the patients was described as asymptomatic; however, in six cases, no information regarding symptoms was available. Most patients were treated surgically by resection (22, 65%), and 13 patients (38%) received adjuvant CTx. LR occurred in six cases (18%) within five months in the mean (range, 48 days–one year). Thirteen patients (25%) showed no distant metastases at the time of diagnosis of ESOS, of which five subsequently developed metastases. Fifteen patients died within six months in the mean; the longest survival was eight years after diagnosis. Six cases of ESOS were only diagnosed on autopsy, pointing out both the aggressiveness and poor prognosis of this entity.

## 4. Discussion

Late metastatic recurrence of OS is uncommon, and visceral metastases, in general, are extremely rare. We performed a retrospective data analysis of OS and visceral metastases to the hepatopancreatobiliary system, combined with a systematic literature review, in order to demonstrate both the infrequent occurrence of visceral involvement and the deductible importance of long-term FU.

Our study is limited by potential language and selection bias, as non-English and non-German studies were translated, possibly leading to a loss of information. The quality of included studies was inconsistent, with some reports providing incomplete data. Despite a comprehensive search strategy, additional published cases may have been missed. Our literature review is likely to detect visceral oligometastasis rather than hepatopancreatobiliary lesions in multimetastatic stages, as the latter are less frequently reported in case series or reviews. Our retrospective data analysis is limited by potential information bias, as non-digitized historical medical records lack imaging data and are solely based on written medical reports, which may be incomplete or inconsistent. Data were not originally retrieved for research purposes, introducing a risk of misclassification and limited availability. Only two of the included patients had histopathological confirmed visceral metastases from OS; the diagnosis of the remaining four patients relied on imaging since visceral metastasis occurred in advanced stages and biopsy was forgone in multimetastatic disease. Therefore, a further histopathological work-up of the visceral metastatic tissue was only possible for patient 1, as tissue is stored for no longer than 30 years at our institution. Before 1990, diagnostics and FU were not as systematic as nowadays; thus, potential visceral metastasis may not have been found. All visceral metastases were detected after 1990, and only two out of six patients received their initial OS diagnosis before 2000. However, since ESOS in the pancreaticobiliary system are typically treated by other departments, these rare cases are unlikely to be entered into the registry.

To our knowledge, there are only 51 published cases of visceral metastases from conventional OS to the hepatopancreatobiliary system. With six cases, our series is the largest single-center dataset compared to date, since only case reports are available. This underlines the rarity of this entity. It should be noted that pathohistological confirmation is not given in all cases, neither in our cohort nor in the literature.

The mean age of 32 years at visceral metastasis from conventional OS in the literature corresponds to our retrospective data analysis. The mean interval of three years between diagnosis of OS and occurrence of visceral metastases is shorter compared to our patient cohort, with an interval of more than five years. The majority of published reports involved pancreatic lesions, whereas we identified only one such case (patient 1). The remaining cases affected the liver. At only 16 mm, the detected metastasis in patient 1 is significantly smaller than the mean pancreatic lesion size of 58 mm reported in the literature. The mean hepatic lesion size of 44 mm in our analysis equals the reported size in the literature.

There are only eight published cases [31,32,34,48,56,57,58,61] with hepatopancreatobiliary lesions as the first site of metastatic disease. In contrast to patient 1, metastasis occurred only within two years after diagnosis, making our patient’s story extremely unique. Longer metastasis intervals exceeding ten years have been described twice, but both patients already had additional metastatic sites at the time of diagnosis [37,46]. The case of patient 1 is exceptional, with the development of a solitary pancreatic metastasis more than ten years after initial diagnosis and without other metastatic sites or LR. To our knowledge, this is the longest reported interval for a solitary visceral metastasis of conventional OS. This patient’s case underlines the importance of long-term oncologic surveillance in OS patients, even in extended disease-free intervals. In different tumor entities, including sarcoma, oligometastasis to the pancreas occurs rarely, typically with a long disease-free interval, and can be treated by surgical resection with good survival results, dependent on the tumor entity [91]. Common FU guidelines [26,27,28] with local MRI or CT scans and chest imaging every two to four months in the first three years after completion of initial treatment, every six months until the sixth year, and annually thereafter may be helpful for early detection of LR or pulmonary metastases. However, visceral metastasis might still be overlooked. Since our FU program ends after ten years of surveillance, the pancreatic lesion would not have been detected that early without the FDG-PET-CT scan that was performed due to the periprosthetic joint infection. This multiphase, contrast-enhanced technique facilitates the detection of subtle visceral metastases compared with low-resolution or non-contrast protocols [92,93]. The associated radiation exposure should be considered in the context of repeated surveillance imaging, though the CT component is the dominant dose contributor [94,95]. Given the fact that the lesion was described already ten months before, this case emphasizes the necessity of considering late-onset solitary metastases in atypical locations, which may initially appear benign. Multidisciplinary management plays a critical role in such cases, particularly in patients with slow tumor progression and no systemic disease.

Liquid biopsy, particularly through the analysis of circulating tumor DNA (ctDNA), was shown to detect early relapses and disease progression in OS [96,97]. However, at present, no studies have reported a FU exceeding five to ten years; therefore, its utility specifically for detecting very late-onset metastases remains unestablished. Theoretically, these minimally invasive approaches could enable long-term surveillance and earlier diagnosis of visceral recurrences, potentially improving patient management and outcomes.

Overall survival in our small cohort of six patients was 50% and 33% at five and ten years, hence, this was evidently lower compared to our general findings in a large-scale series of OS of the extremities [98] with an overall survival of 72% and 70% at five and ten years. However, with a limited number of patients with hepatopancreatobiliary metastases, we found a statistical comparison unjustified in the face of considerable selection bias on the one hand, and clearly different clinical outcomes on the other. Therefore, it also seems hard to deduce clear implications for different FU management procedures in these patients.

Compared to diffuse metastasis, oligometastatic visceral lesions of OS are even more uncommon, with only 0.2% of our cohort. This extraordinary rarity may not justify a change in FU, as formal recommendations for long-term imaging or risk stratification currently do not exist. While theoretically, extended imaging or ctDNA monitoring could allow earlier detection, the very low incidence, potential radiation exposure, and lack of cost-effectiveness data make such strategies difficult to establish. Future studies are required to explore whether these approaches might complement a traditional FU, but to date, no general recommendations can be made for the surveillance of rare, yet possible, visceral metastases. In our current clinical practice, we recommend a FU regimen, as described in the Materials and Methods section, with chest and abdominal CT scans as well as local MRI every four months during the years 1–3, subsequently every six months, and annually from year 6 onwards until year 10. For a FU exceeding ten years, we discuss the aforementioned issues with the patient and continue a yearly restaging upon individual preference.

## 5. Conclusions

This study highlights the need for a vigilant, long-term FU. In particular, the rare case of late metastatic occurrence of OS in the pancreatic tail ten years after initial diagnosis underlines the importance of considering late-onset solitary metastases in atypical locations. Surveillance should continue for at least ten years, especially in high-grade lesions. In terms of very late recurrent disease and the detection of rare visceral metastases, a reconsideration of long-term surveillance strategies might be appropriate.

## Figures and Tables

**Table 1:** Overview of patient cohort.

Pat.	Age at Visceral Metastasis	Sex	Type	Location	Treatment of Primary	Interval (OS Diag to Detection of Visceral Met) (Months)	Symptoms	Metastatic Site	Single/ Multiple	Size (mm)	Calcification in Imaging	Treatment	Local and Distant Relapse	Outcome	FU/Survival Since Visceral Met (Months)
1	35	m	osteoblastic (G3)	distal femur	CTx, OP, CTx (EURAMOS)	129	none	pancreas	single	16	yes	left pancreatectomy with splenectomy and gastric wedge resection	none	AWD	8
2	25	m	n/a	proximal humerus	CTx, OP, CTx (COSS-80)	120	recurrent fever episodes, occasional dyspnea	liver	single	120 × 90	n/a	right hemihepatectomy	lung (concurrent with liver met), LR 2 years after diag	DOD	24
3	20	m	chondroblastic (G3)	distal femur	CTx (protocol unknown), OP, dendritic cell therapy, RTx	60	ileus	liver	multiple	n/a	no	none	lung, pleura, bone, heart, soft tissue, adrenal gland, bowel	DOD	0.17
4	21	m	chondroblastic (G3)	distal femur	CTx, OP, CTx (EURAMOS)	35	n/a	liver	multiple	20, 10	no	none	lung, bone, spine, soft tissue	DOD	3
5	33	m	teleangiectatic (G3)	sternum	CTx, OP, CTx (EURAMOS)	9	n/a	liver	single	25 × 20	no	none	lung	DOD	3
6	62	m	osteoblastic (G3)	proximal femur	CTx, OP, CTx (COSS-86-C)	primarily metastasized	none	liver	multiple	n/a	n/a	none	lung, mediastinal, adrenal gland, soft tissue	DOD	2

Abbreviations: Pat., patient; m, male; n/a, not available; CTx, chemotherapy; OP, surgery (resection); RTx, radiotherapy; OS, osteosarcoma; Diag, diagnosis; LR, local recurrence; AWD, alive with disease; DOD, died of disease; “FU/Survival since Visceral Met” describes either FU time (AWD), or time until death (DOD), calculated from the time of diagnosis of visceral involvement; FU, follow-up.

**Table 2:** Distribution of metastases from conventional OS to the hepatopancreatobiliary system.

Case	Sex	Age at Metastasis	Location	Interval (OS Diag to Detection of Visceral Met) (Months)	Symptoms	Metastatic Site	Size (mm)	Treatment	Other Metastases	Outcome	FU/Survival Since Visceral Met (Months)
1 [29]	f	57	proximal femur	15 prior to OS diagnosis	abdominal pain	pancreas tail	25	distal pancreatectomy	skin, bone, brain	DOD	18
2 [5]	f	35	distal tibia	44	none	pancreas body	3	distal pancreatectomy and splenectomy	LR after 17 months, subsequently lung	AWD	48
3 [10]	f	38	n/a	43	abdominal pain, jaundice	pancreas head	57	n/a	tumor thrombus in superior mesenteric vein	n/a	
4 [30]	m	21	femur	n/a	n/a	pancreas body	n/a	n/a	lung, bone	DOD	n/a
5 [30]	m	17	tibia	n/a	n/a	pancreas head (multiple)	n/a	n/a	lung, bone, lymph nodes, chest, kidney	DOD	n/a
6 [30]	f	13	femur	n/a	n/a	pancreas tail	n/a	n/a	lung, bone	DOD	n/a
7 [6]	f	19	distal femur	48	abdominal pain and swelling	pancreas tail	150 (US)	partial resection of the pancreatic tail, drainage of ruptured cyst	lung, jejunum (LR)	DOD	n/a
8 [31]	f	44	proximal fibula	24 postoperative	none	pancreas tail	n/a	distal pancreatectomy	subsequently lung	AWD	24
9 [9]	m	18	proximal tibia	48	abdominal pain, vomiting	pancreas head	45	pancreatoduodenectomy + CTx	lung	AWD	7
10 [3]	f	25	distal femur	77	acute anemia, melena	pancreas, duodenum	n/a	pancreatoduodenectomy + CTx	lung	AWD	11
11 [4]	m	14	spine	18	nausea, vomiting, diarrhea, melena, jaundice	pancreas head, adjacent duodenum	80	n/a	LR	DOD	0.5
12 [32]	f	47	proximal fibula	36	none	pancreas tail	13	distal pancreatectomy + CTx	subsequently lung	AWD	14
13 [33]	f	54	maxilla	24	n/a	pancreas neck and body (multiple)	body 22, neck 30 (EUS)	n/a	n/a	n/a	
14 [34]	m	27	n/a	20	abdominal pain	pancreas body	n/a	resection	none	n/a	
15 [35]	f	53	distal femur	48	abdominal pain, fullness, early satiety	pancreas genu, body, and tail, adjacent gallbladder fossa, duodenum, and liver hilum	pancreas 110 (EUS)	drainage	lung	AWD	n/a
16 [36]	f	33	maxillary sinus	42	n/a	pancreas	n/a	CTx	lung	AWD	5
17 [37]	f	10	distal femur	18	n/a	pancreas, liver (single)	n/a	laparotomy + CTx	lung, kidney	DOD	2
18 [37]	f	25	distal femur	132	n/a	pancreas tail	n/a	resection	lung	AWD	7
19 [13]	f	13	fibula	14	pyloric stenosis symptoms, vomiting	pancreas head, adjacent duodenum, liver (single)	pancreas 60	pancreatoduodenectomy, chemoembolization of hepatic lesions	lung	DOD	16
20 [38]	m	60	proximal femur	36	abdominal pain, jaundice	pancreas head, subsequently liver (multiple)	pancreas 25, liver < 20	n/a	n/a	n/a	
21 [38]	f	39	distal femur	24	abdominal pain	liver	unmeasurable (US)	n/a	lymph nodes paratracheal, small bowel	n/a	
22 [39]	f	69	proximal femur	36	jaundice	pancreas head, subsequently liver (multiple)	n/a	cholecystojejunostomy	lung, bone	n/a	
23 [40]	m	20	distal femur	24	none	pancreas tail	68 (MRCP)	en-bloc resection with distal subtotal gastrectomy, en-bloc spleen-preserving distal pancreatectomy, en-bloc resection of transverse mesocolon and Roux-en-Y gastrojejunostomy reconstruction	lung	n/a	
24 [41]	f	63	distal femur	n/a	n/a	pancreas head, subsequently adjacent duodenum	19	n/a	lung, brain	DOD	n/a
25 [42]	f	19	distal femur	66	n/a	pancreas, adjacent duodenum, subsequently liver	50	pancreatoduodenectomy + CTx	lung, subsequently peritoneum	DOD	25
26 [43]	f	49	distal femur	24	back pain	pancreas head	70	palliative RTx	lung, bone	DOD	2
27 [43]	f	42	distal femur	6	abdominal and back pain	pancreas tail	n/a	palliative RTx	lung	DOD	1
28 [11]	m	15	femur	13	none, gradually abdominal pain	liver, subsequently pancreas	n/a	none	lung, subsequently pancreas	DOD	2
29 [11]	m	50	tibia	17	abdominal distension	liver	n/a	none	lung, brain	DOD	6
30 [11]	m	54	clavicula	31	liver dysfunction, subsequently diabetes	liver, subsequently pancreas	n/a	none	lung, bone, subsequently pancreas	AWD	4
31 [44]	m	45	distal femur	27	abdominal pain, fever, nausea, loose stools, decreased appetite, insomnia	liver (multiple)	n/a	n/a	lung, LR, colon, retroperitoneal space, adrenal gland, paracolic	DOC	1
32 [45]	m	24	tibia	42	abdominal pain, nausea, vomiting, anorexia	liver (multiple)	20–50 (OP)	partial hepatectomy, distal jejunum, and proximal ilium resection	lung, small bowel	n/a	
33 [12]	m	32	tibia	66	acute anemia, melaena, hematemesis	duodenum, adjacent pancreas head	90 (OP)	gastroduodenopancreatectomy, cholecystectomy	lung	DOC	2
34 [46]	m	37	femur	204	n/a	pancreas	n/a	n/a	lung, brain, subsequently pancreas, and adrenal gland	n/a	
35 [47]	f	26	3^rd^ rib	12	abdominal distension	liver (single)	n/a	n/a	lung	DOD	6
36 [48]	m	27	femur	48	anorexia, nausea, vomiting, iron-deficiency anemia	duodenal papilla	40 (Endoscopy)	n/a	none	n/a	
37 [49]	m	19	distal femur	29	abdominal pain, vomiting, weight loss, symptoms of intestinal obstruction	liver (multiple)	n/a	none (refused CTx)	peritoneum, abdominal	DOD	n/a
38 [50]	f	28	proximal humerus	6 postoperative	n/a	liver (single)	n/a	none (best supportive care)	lung, LR (positive margins)	AWD	n/a
39 [51]	m	21	proximal tibia	50	n/a	liver	n/a	CTx	lung, bone, retroperitoneal, subsequently omentum	DOC	6
40 [52]	m	24	proximal tibia	primarily metastasized	n/a	liver (multiple)	n/a	CTx	lungs, mediastinum, later bone	DOD	5
41 [53]	m	47	proximal tibia	13 postoperative	n/a	liver (multiple)	n/a	n/a	lymph nodes, soft tissue, L5, pelvic bone, iliac psoas muscle	DOC	11
42 [7]	m	39	humerus	primarily metastasized	n/a	liver (single)	25 (2 months after diag)	RFA	lung, kidney	DOD	13
43 [54]	n/a	15	distal femur	primarily metastasized	n/a	liver (multiple)	n/a	n/a	lung, bone, brain	DOD	7–8 (after diag)
44 [55]	f	16	femur	7	n/a	liver (single)	n/a	CTx	lung	n/a	
45 [56]	m	16	proximal humerus	19 after EOT	abdominal pain, fever	liver (multiple)	162	n/a	none	DOD	3
46 [57]	f	36	tibia	36	abdominal pain	liver (single)	37	RTx	none	AWD	3
47 [58]	f	36	distal femur	primarily metastasized	n/a	liver (multiple)	35, 9, 7	resection planned after CTx	none	AWD	5
48 [59]	f	23	femur	84	n/a	pancreas	170	pancreatoduodenectomy	lung, LR in pancreatic tail 24 months postoperatively, died 9 months later	DOD	33
49 [60]	m	21	tibia	during 3rd cycle of adjuvant CTx	abdominal pain	pancreas head	13 (US)	continuation of ongoing CTx	lung	AWD	n/a
50 [14]	f	30	proximal tibia	84	abdominal pain and distension, vomiting, lack of intestinal transit	liver (multiple)	6	resection	lung, subsequently brain, bowel	n/a	
51 [61]	F	14	humerus	0.3	abdominal pain	liver (single)	100 (X-ray, 1.5 months after diag)	antimitotic treatment with intramuscular injections of Endoxan	none	DOD	5

Abbreviations: n/a, not available; f, female; m, male; OS, osteosarcoma; Diag, diagnosis; CTx, chemotherapy; RTx, radiotherapy; RFA, radiofrequency ablation; LR, local recurrence; DOD, died of disease; AWD, alive with disease; DOC, died of other cause; Size (mm), lesion size in mm (largest diameter) from CT imaging as not other indicated; other than that from ultrasound (US), endosonographic ultrasound (EUS), MRCP (magnetic resonance cholangiopancreatography), endoscopy or intraoperatively (OP); “FU/Survival since Visceral Met” describes either the FU time (AWD), or the time until death of disease (DOD) or other cause (DOC), calculated from the time of diagnosis of visceral involvement; FU, follow-up.

**Table 3:** ESOS of the hepatopancreatobiliary system.

Case	Sex	Age at Diag	Site	Symptoms	Size (mm)	Treatment	Local and Distant Relapse	Outcome	FU/Survival (Months)
1 [62]	m	52	liver	fatigue, back pain, meteorism	n/a	none (autopsy)	heart, small bowel	DOD	1.6
2 [23]	m	73	liver	abdominal pain, discomfort	170	right hepatectomy	LR after 48 days	DOD	n/a
3 [21]	m	59	liver	abdominal pain	120	right hepatectomy	none	AWD	n/a
4 [19]	m	62	liver	abdominal pain, fever	78	partial hepatic lobectomy	none	AWD	n/a
5 [22]	m	70	liver	abdominal discomfort, fever	92 (US)	CTx	none	DOD	4
6 [63]	m	72	liver/ pancreatic head/ duodenum	abdominal pain, distension, weight loss, loss of appetite	250 *	none (autopsy)	none	DOD	4
7 [64]	m	50	liver	abdominal pain	48	right hemihepatectomy	none	DOD	2
8 [65]	m	67	liver	swelling	100 *	CTx (autopsy)	n/a	DOD	n/a
9 [66]	m	72	liver	abdominal pain, loss of appetite, weight gain	130 *	none (autopsy)	lung	DOD	0.2
10 [67]	m	75	liver	icterus, weight loss	50	none (autopsy)	omentum majus	DOD	1
11 [68]	f	19	liver	abdominal discomfort, early satiety, anorexia	150 (MRI)	left hepatic lobectomy with en-bloc cholecystectomy	none	AWD	36
12 [69]	m	64	liver	abdominal distension and colic, constipation	130 *	none (autopsy)	none	DOD	0.7
13 [70]	m	71	liver	abdominal pain, signs of bowel obstruction	250	resection	subsequently peritoneum, stomach, bowel, and lung	DOD	2
14 [71]	f	69	liver	dyspnea, right pleuritic chest pain, intermittent fever	150	hemihepatectomy with concomitant partial resection of the parietal peritoneum of the anterior abdominal wall and diaphragm	LR after 2 months	DOD	2 (postoperatively)
15 [72]	m	45	liver/ adrenal gland/ portal vein	abdominal pain, discomfort	270	hepatic trisegmentectomy (right lobe and medial segment of the left lobe) and cholecystectomy	none	AWD	0.7
16 [73]	f	73	liver	abdominal pain	n/a	n/a	n/a	n/a	
17 [18]	m	47	liver	abdominal pain	110	right hepatic lobectomy	n/a	AWD	3
18 [74]	m	79	liver	abdominal pain, bloating	171	resection; transarterial chemoembolization after LR	LR after 1.5 months	DOD	2 (after LR)
19 [75]	m	65	liver	abdominal pain, bloating, nausea	n/a	none	lymph nodes, lung, Th3	n/a	
20 [76]	f	61	liver	n/a	75	resection	LR after 7 months, lung	DOD	1.5 (after LR)
21 [77]	f	66	liver	abdominal pain	n/a	liver transplantation	subsequently, pleura, lymph nodes, peritoneum, abdominal cavity	n/a	
22 [78]	m	50	pancreas/ left kidney	back pain	n/a	distal pancreatectomy with partial gastrectomy, splenectomy, left nephrectomy	LR after 6 months, subsequently the renal bed, hepatogastric area, lymph nodes, lung, and peritoneum	DOD	20
23 [79]	f	56	pancreatic tail	n/a	27	distal pancreatectomy with splenectomy + CTx; RTx planned	subsequently scalp	n/a	
24 [80]	f	75	duodenum/ pancreatic head	fatigue, progressive muscle weakness, melena	54	pylorus-preserving pancreatoduodenectomy	subsequently liver (size 150 mm)	n/a	
25 [81]	f	61	gallbladder	abdominal discomfort, nausea, subcostal tenderness	n/a	cholecystectomy	subsequently abdominal wall, peritoneal cavity, liver, intestines, omentum majus and minus, mesentery, and lung	DOD	9
26 [82]	f	74	retroperitoneum/ left kidney/ pancreatic tail	flank pain, abdominal distension	160	chemoembolization	n/a	DOC	18
27 [83]	f	72	gallbladder/liver	abdominal pain, weight loss, loss of appetite	45	cholecystectomy with partial CBD excision	none	AWD	n/a
28 [84]	f	45	gallbladder	abdominal discomfort	50	cholecystectomy	none	AWD	16
29 [85]	m	64	pancreas	abdominal pain, jaundice, weight loss	70	whipple procedure	none	AWD	3.5
30 [86]	f	44	pancreas	abdominal discomfort	100 (US)	splenectomy, removal of pancreatic tail, left renal capsule, and surrounding lymph nodes	LR after 1 year	n/a	
31 [87]	m	66	liver	abdominal pain	60	CTx	lymph nodes	DOD	6
32 [88]	m	60	duodenum	abdominal pain, anemia, melena, fatigue	n/a	duodenopancreatectomy	none	AWD	96
33 [89]	f	50	gallbladder	abdominal pain	35 (OP)	cholecystectomy	none	AWD	6
34 [90]	f	62	gallbladder	abdominal pain, weight loss	120 (OP)	cholecystectomy	n/a	DOD	14

Abbreviations: n/a, not available; f, female; m, male; diag, diagnosis; CTx, chemotherapy; RTx, radiotherapy; LR, local recurrence; DOD, died of disease; AWD, alive with disease; DOC, died of other cause; Size (mm), lesion size in mm (largest diameter), in CT imaging as not other specified; other than that in ultrasound (US) or MRI imaging, on surgical (OP) or histological examination, e.g., autopsy (*); CBD, common bile duct; “FU/Survival” describes either the FU time (AWD,) or the time until death of disease (DOD) or other cause (DOC), calculated from the time of diagnosis; FU, follow-up.

## Data Availability

The original contributions presented in this study are included in the article. Further inquiries can be directed to the corresponding author.

## Abbreviations

The following abbreviations are used in this manuscript:

AWD	Alive with Disease
BSG	British Sarcoma Group
CBD	Common Bile Duct
CT	Computer Tomography
ctDNA	Circulating tumor DNA
CTx	Chemotherapy
Diag	Diagnosis
DLP	Dose-length product
DOC	Died of other Cause
DOD	Died of Disease
ESMO	European Society for Medical Oncology
ESOS	Extraskeletal Osteosarcoma
EUS	Endosonographic Ultrasound
f	Female
FDG	FlDeoxyGlucose
FU	Follow-up
HE	hematoxylin–eosin
LR	Local recurrence
m	Male
mm	Millimeter
MRCP	Magnetic Resonance Cholangiopancreatography
MRI	Magnetic Resonance Imaging
n/a	Not available
NCCN	National Comprehensive Cancer Network
OP	Surgery
OS	Osteosarcoma
Pat.	Patient
PET	Positron Emission Tomography
RFA	Radiofrequency Ablation
RTx	Radiotherapy
US	Ultrasound
